# Driving Under the Influence of Alcohol among Road Traffic Accident Patients Presenting to a Tertiary Care Centre

**DOI:** 10.31729/jnma.8260

**Published:** 2023-09-30

**Authors:** Pramod Joshi, Mahesh Karmacharya, Sailendra Kumar Duwal Shrestha

**Affiliations:** 1 Department of Orthopedics, National Trauma Center, Mahankal, Kathmandu, Nepal; 2 Department of Orthopedics, Nepal Armed Police Force Hospital, Balambu, Kathmandu, Nepal

**Keywords:** *accidents*, *alcohol consumption*, *bone*, *Nepal*, *trauma*

## Abstract

**Introduction:**

Driving under the influence is one of the most significant risk factors for road traffic accidents, leading to severe and multiple orthopaedic injuries. The aim of this study was to find out the prevalence of driving under the influence of alcohol among road traffic accident patients presenting to a tertiary care centre.

**Methods:**

A descriptive cross-sectional study was conducted on patients involved in road traffic accidents presenting to a tertiary care centre. Data from 10 January 2020 to 9 December 2021 were collected between 22 July 2023 to 22 August 2023 from the hospital records after receiving ethical approval from the Institutional Review Committee. Patients who had road traffic accidents and were diagnosed with fractures were included in the study. Patients with incomplete hospital records were excluded from the study. Convenience sampling method was used. The point estimate was calculated at a 95% Confidence Interval.

**Results:**

Among 2,519 patients, 307 (12.19%) (10.91-13.47, 95% Confidence Interval) were driving under the influence of alcohol when involved in a road traffic accident. Out of 307 patients, 305 (99.35%) accidents involved two-wheelers. A total of 118 (38.44%) patients suffered soft tissue injuries, while 47 (15.31%) sustained head injuries, and 28 (9.12%) sustained facial injuries.

**Conclusions:**

The prevalence of driving under the influence of alcohol among road traffic accident patients presenting to a tertiary care centre was similar to other studies done in similar settings.

## INTRODUCTION

According to the World Health Organization (WHO), road traffic accidents (RTA) were one of the leading causes of injuries with 2.34 million in 2020 from 0.99 million in 1990, causing around 25% of all injuries leading to death.^1,2^ The primary cause of RTA-related injuries and mortalities, other than vehicle, road, and environment, is drunk driving, also known as driving under the influence.^3^ A staggering 41% of traffic fatalities are caused by driving under the influence in the United States, 32% in the European Union, and 59% in India.^4,5^

In Nepal, driving under the influence is one of the most common causes of RTA. Nonetheless, the scale of the problem is under-recognized and concealed because of a lack of high-quality research data.

The aim of this study was to find out the prevalence of driving under the influence of alcohol among road traffic accident patients presenting to a tertiary care centre.

## METHODS

This descriptive cross-sectional study was conducted among patients involved in road traffic accident admitted to the National Trauma Center, Mahankal, Kathmandu, Nepal. Data from 10 January 2020 to 9 December 2021 were collected between 22 July 2023 to 22 August 2023 from the hospital records after receiving ethical approval from the Institutional Review Committee (Reference number: 37/2080/81). Patients who had RTA and were diagnosed with fractures were included in the study. Patients with incomplete hospital records were excluded from the study. Convenience sampling method was used. The sample size was calculated by using the following formula:


$$\begin{array}{ll} \text{n} & ={\text{Z}}^{2}\times \frac{\text{p}\times \text{q}}{{\text{e}}^{\text{2}}}\\ & =1.96^{2}\times \frac{0.50\times 0.50}{0.02^{2}}\\ & =2401 \end{array}$$

Where,

n = minimum required sample size
Z = 1.96 at 95% Confidence Interval (CI)
p = prevalence taken as 50% for maximum sample size calculation
q = 1-p
e = margin of error, 2%

The minimum required sample size was 2401. However, the final sample size taken was 2,519. Patients with RTA while driving under the influence were confirmed during police verification at the time of the accident. During police verification, patients who had an alcohol concentration greater than 0.22 mg/l were confirmed to be involved in alcohol-related RTA.^6^ Patients or their relatives provided information concerning the type of vehicle and their involvement as a rider, passenger, or pedestrian during RTA. Patients who presented with orthopaedic fractures were sent for a plain film X-ray for a confirmatory diagnosis. Confirmation for the diagnosis of the fracture and its severity after a review of the images was based on an X-ray. A polytrauma was defined as two or more severe injuries in more than one area of the body, while a multiple injury was defined as two or more serious injuries in one area.^7^

Data was extracted using the extraction sheet using Microsoft Excel 2016 and analysed using IBM SPSS Statistics version 17.0. The point estimate was calculated at a 95% Confidence Interval.

## RESULTS

Among 2,519 patients, 307 (12.19%) (10.91-13.47, 95% CI) were driving under the influence of alcohol when involved in a road traffic accident. The median age of patients was 28 years. There were 303 (98.70%) males, 4 (1.30%) females (Table 1).

**Table 1:** Demographic characteristics (n = 307).

Characteristics	n (%)
**Age group (years)**	
16-20	40 (13.03)
21-30	163 (53.09)
31-40	64 (20.85)
41-50	32 (10.42)
51-60	4 (1.30)
≥61	4 (1.30)
**Gender**	
Male	303 (98.70)
Female	4 (1.30)

Among 307 patients, 224 (72.96%) were from Bagmati province, while 33 (10.75%) and 19 (6.19%) were from Madhesh and Koshi province, respectively. There were 101 (32.90%) patients from Kathmandu district, 21 (6.84%) from Dhading and 12 (3.91%) from Ramechhap. There were 121 (39.41%) alcohol-related RTA at 21:00 p.m. to 00:00 a.m., while 95 (30.94%) at 00:01-03:00 a.m.

A total of 305 (99.35%) accidents were in two-wheelers and 2 (0.65%) in four-wheelers. A total of 117 (38.11%) had soft tissue injuries, while 47 (15.31%) and 28 (9.12%) had head and facial injuries (Table 2).

**Table 2:** Types of injury (n= 307).

Diagnosis	Injury	Two-wheeler n (%)	Four-wheeler n (%)
Upper limb fractures	Shoulder dislocation	2 (0.65)	-
	Humerus fracture	3 (0.98)	-
	Radius ulna fracture	6 (1.95)	-
	Radius fracture	9 (2.93)	-
	Metacarpal fractures	7 (2.28)	-
	Phalanx fracture	7 (2.28)	-
Lower limb fractures	Femur fracture	11 (3.58)	1 (0.33)
	Tibia fibula fracture	6 (1.57)	-
	Tibia fracture	11 (3.58)	-
	Malleoli fracture	2 (0.65)	-
	Metatarsal fracture	2 (0.65)	-
Additional fractures	Head injury	47 (15.31)	-
	Facial injury	28 (9.12)	-
	Clavicle fracture	10 (3.26)	-
	Spinal injury	10 (3.26)	-
	Chest injury	8(2.61)	-
	Abdominal trauma	6 (1.57)	-
	Soft tissue injury	117 (38.11)	1 (0.33)
	Crush injury	2 (0.65)	-
	Others^*^	11 (3.58)	-

* Fractures, such as cuneiform, patella, ulnar, ribs, calcaneum, and fibula; dislocations, such as knee, hip, elbow, interphalangeal joint; injuries, such as neck and brachial plexus.

Two-wheelers had 37 (12.05%) and 64 (20.85%) incidences of multiple injuries and polytrauma respectively (Table 3).

**Table 3:** Traumatic severity (n= 307).

Severity	Two-wheeler n (%)	Four-wheeler n (%)
Single injury	204 (66.45)	-
Multiple injuries	37 (12.05)	2 (0.65)
Polytrauma	64 (20.85)	-

## DISCUSSION

The prevalence of driving under the influence of alcohol in this study was 307 (12.19%), similar to the finding of another study which was 15%.^8^ A systematic review of alcohol consumption among RTI patients reported a similar prevalence of 15%, while several previous Indian studies reported a varying prevalence of alcohol-related RTA (14-23%).^9^ Despite the strict traffic regulations, a study conducted in Kathmandu, the capital city of Nepal, found an increase of 1.5-fold in crashes in RTA during a fiscal year.^10^

In this study, the highest incidences of alcohol-related RTA were among the 21-30 years age group 163 (53.09%). Studies across different parts of the world (including Nepal) have also reported that people between the ages of 21-40 are more likely to experience RTA, with incidences ranging from 27 to 71%.^10-13^ These significant numbers of RTA among adults reflect substantial losses of the most productive age group worldwide each year and can be attributed to more frequent travel for employment, education, and economic pursuits, as well as a moral proclivity towards breaking traffic laws or driving recklessly.^11,12^

In our study, there was an approximately 76-fold greater prevalence of alcohol-related RTA among men than among women. It is higher than the findings of another study from Nepal that men (78.31%) have more RTA than women (21.69%).^10^ RTA have also been reported to be more common in males (83-85%) than in females (15-17%) as summarized in a study from India.^13^ The higher male incidence of alcohol-related RTA may be due to men's higher involvement in various social and economic activities as well as traffic violations (driving while tired, speeding, drink-driving, and driving without a helmet or seatbelt), as well as social limitations regarding women's riding habits and cautious driving.^10,12^ The median time of alcohol-related RTA in this study was 9:23 p.m., with most incidents occurring from 9:00-12:00 p.m., followed by 0:01-3:00 a.m. A study conducted in Nepal reported a higher number of RTA during the day from 12-3 p.m.^8^

In this study, alcohol-related RTA were more common in two-wheelers (99.35%) than four-wheelers (0.65%), consistent with the results of a Nepalese study.^8^ Similarly, in a study two-wheeler riders' fracture risk was 150-fold greater than four-wheeler riders.^9^

The most common injury sustained in this study involved soft tissue, 118 (38.44%). Besides, only two-wheeler RTA resulted in fractured upper limbs (11.07%) and clavicles (3.26%), as well as injured chests (2.61%) and faces and abdomen (11.07%). Similar study reported that external injuries tend to occur on the upper and lower limbs, as well as on the head and face.^14^

Approximately two-thirds of the victims in this study suffered single injuries, while one-fifth sustained polytrauma, and one-eighth sustained multiple injuries. There is a possibility that high rates of polytrauma may have been caused by victims being directly exposed to disproportionately intense collisions inside vehicles, or by large percentages of drivers' not wearing seatbelts.^15^

This study has several limitations. There is the possibility that the study underestimated the actual prevalence of RTA because it focused exclusively on alcohol-related RTA. As our study is single-centered therefore, these results may be overestimated or underestimated.

## CONCLUSIONS

The prevalence of driving under the influence of alcohol among road traffic accident patients presenting to a tertiary care centre was similar to other studies done in similar settings. This evidence can be used to support interventions to formulate stronger enforcement of existing drink-driving countermeasures in reducing alcohol-related RTIs.
